# Oxidative Stress and Histological Changes in a Model of Retinal Phototoxicity in Rabbits

**DOI:** 10.1155/2014/637137

**Published:** 2014-05-27

**Authors:** Manuel Saenz-de-Viteri, Henar Heras-Mulero, Patricia Fernández-Robredo, Sergio Recalde, María Hernández, Nicholas Reiter, Maite Moreno-Orduña, Alfredo García-Layana

**Affiliations:** ^1^Experimental Ophthalmology Laboratory, School of Medicine, University of Navarra, 1 Irunlarrea Street, 31008 Pamplona, Spain; ^2^Department of Ophthalmology, Clínica Universidad de Navarra, School of Medicine, University of Navarra, 36 Pio XII Avenue, 31008 Pamplona, Spain

## Abstract

Photochemical damage occurs after an exposure to high energy radiation within the visible spectrum of light, causing morphological changes in the retina and the formation of superoxide anion. In this study we created a model of phototoxicity in rabbits. Animals were exposed to a light source for 120 minutes and were sacrificed immediately or one week after exposure. Outer nuclear layer and neurosensory retina thickness measurements and photoreceptor counting were performed. Caspase-1 and caspase-3 were assessed by immunohistochemistry. Dihydroethidium was used to evaluate in situ generation of superoxide and thiobarbituric acid reactive substances were measured in retinal homogenates as indicators of lipid peroxidation. The total antioxidant capacity and oxidative ratio were also determined. Retinas from rabbits exposed to light showed higher levels of lipid peroxidation than the unexposed animals and a decrease in outer nuclear layer and neurosensory retina thickness. Our study demonstrates that light damage produces an increase in retinal oxidative stress immediately after light exposure that decreases one week after exposure. However, some morphological alterations appear days after light exposure including apoptotic phenomena. This model may be useful in the future to study the protective effect of antioxidant substances or new intraocular lenses with yellow filters.

## 1. Introduction

Photochemical damage occurs after an exposure to high energy radiation with a wavelength within the visible spectrum of light. It has been demonstrated that the blue portion of the visible spectrum of light is the most harmful, producing disturbances of the outer blood retinal barrier in the retinal pigmented epithelium (RPE) [1]. At the retinal level, exposure to light causes an increase in phagocytosis of photoreceptor outer segments (POS) [2] and induces the formation of superoxide anion by the RPE [3].

Tissues with a high proportion of membrane lipids and a high tissue oxygen concentration are most sensitive to damage by oxidative stress [4]. The retina has a high oxygen tension (70 mmHg) which makes it very vulnerable to oxidative stress [5]. Moreover, the retina and more specifically the POS possess very high levels of polyunsaturated fatty acids which further increases the sensitivity to oxidative damage and lipid peroxidation of cell membranes [6, 7] as well as phenomena of cell death (apoptosis or necrosis) [8].

Evidence suggests that excessive light exposure plays an important role in the development and progression of age related macular degeneration (AMD) [9–13]. Lipofuscin, a target molecule for phototoxic damage, accumulates in the retina with age, making elderly people more susceptible to light damage [9]. On the contrary, the human lens accumulates yellow chromophores with aging that reduce the transmission of blue and UV light to the retina [9, 10]. However, removal of the lens by cataract surgery, a common procedure in the elderly, restores the amount of visible radiation that is incident upon the retina [13].

Various experimental studies have demonstrated that the retina can be damaged by the effect of light in different animal models [12–14] showing various morphological patterns. Furthermore, these patterns may vary according to species and the severity of damage [15]. Retinal phototoxicity models in small rodents have been the subject of most studies, rather than in other animals. Primary damage occurring in the retinas of rats exposed to white light lies in the outer nuclear layer (ONL) [16] although some damage can be observed in the inner nuclear layer (INL) [17]. In rats and mice, the rods are more sensitive than cones to damage by light [18] while in chickens and pigeons cones are damaged first [19].

Although these models are widely used, they have several problems. One of them is the small eye size that makes them unsuitable for some experimental surgery procedures like cataract surgery and intraocular lens (IOL) implantation.

This is important since some of the most frequently used IOLs have yellow filters in order to protect the retina from harmful blue light, aimed at preventing oxidative stress related diseases such as AMD [12, 13]. Larger animals have also been used. Messner et al. [20] conducted a study in newborn monkeys (*Macaca arctoides*) continuously exposed to a fluorescent light source (400 foot-candles) for periods of 12 hours, 24 hours, 3 days, and 7 days. These authors were able to show structural damage in the retina of the exposed animals, especially evident in the ONL. Other authors have used pigs as experimental animals. Sisson et al. [21] showed how retinal newborn pigs exposed for 72 hours to a source of blue light also suffered extensive damage to retinal cytoarchitecture with vacuolization of photoreceptors and the presence of pyknotic nuclei in the ONL. However, maintenance expenses of these animals are high and availability is much lower.

We used rabbits given that they are more accessible, easier to handle than pigs or nonhuman primates, and their eye size allows performing therapeutic or surgical procedures such as the insertion of IOLs with protective filters [22]. Although studies in rabbits have shown histological changes and dysfunction of the RPE after light exposure [1, 23], photochemical retinal damage in rabbits has not been fully described. In this work, we investigate the effect of photochemical retinal damage on lipid peroxidation and structural modifications in the rabbit retina.

## 2. Methods

### 2.1. Animal Model

Animals were handled according to the rules of the Association for Research in Vision and Ophthalmology (ARVO) and all experiments were approved by the Ethical Committee for Animal Experimentation of the University of Navarra. We used 71 New Zealand white rabbits. Albino rabbits were chosen because of the absence of melanin in the RPE which was supposed to increase retinal susceptibility to phototoxic damage [16]. All specimens were adult females weighing between 2.5 and 3 kg at the beginning of the experiment.

### 2.2. Induction of Phototoxicity

To ensure the absence of basal retinal pathology, the fundus of all animals was explored using a Canon retinography camera (Canon 8 CF 604 retinography camera, Japan), after pupil dilation with tropicamide 1% (Alcon cusí, Barcelona, Spain) and phenylephrine 10% (Alcon cusí, Barcelona, Spain) eye drops. Before light exposure, rabbits were anesthetized by intramuscular injection of ketamine (1 mL/kg) and xylazine (0.5 mL/kg) which was maintained during all exposure. Eyes remained open by placing blepharostat and the cornea was irrigated with saline using an anterior chamber cannula. The phototoxicity model was created with a 150 W white light fibre optic halogen lamp (type 6423 FO. 150 W Philips) with two optical fiber sources through which the light was transmitted that were placed at a distance of 0.5–1 cm from the cornea. Thermal damage was ruled out given that the temperature measured at 0.5 cm from the light source after 30 minutes had only increased by 0.5°C.

Animals were divided into two interventional groups and one control group. Each animal was randomly assigned to different study groups. The 142 eyes of 71 rabbits were distributed into the following groups: control group (C), 120 minutes of light exposure and immediate sacrifice of the animal after exposure (LE), and 120 minutes of light exposure (LEW) with sacrifice of the animal one week after exposure. After anesthesia with 1 mL/kg body weight of ketamine and 0.5 mL/kg body weight of xylazine, rabbits were sacrificed by intravenous injection of T61 (Intervet Deutschland GmbH, Unterschleißheim, Germany) for histological evaluation.

### 2.3. Extraction and Processing of the Retina

Once the animal was sacrificed the eyes were enucleated. Briefly, a 360° peritomy was performed; extraocular muscles, the optic nerve, and vessels were cut. The ocular surface was cleaned of any traces of conjunctiva and washed with saline.

For biochemical analysis purposes, the eyeball was placed on blotting paper and an incision was made 5 mm behind the* limbus* to separate the anterior and posterior poles. Approximately 0.5 mL of vitreous was collected using a 1 mL syringe. The retina was then detached from EPR-choroid complex using forceps and a scalpel blade. Once the samples were removed, they were placed in a 1.5 mL microtube and homogenization was performed with an Ultra-Turrax (IKA T10basic, Staufen, Germany). The samples were divided into aliquots which were kept frozen at −80°C until use.

### 2.4. Preparation of Tissues for Light Microscopy and Conventional Hematoxylin-Eosin Staining

For histological purposes, the standard procedure was applied [24]. Prior to fixation, globes were marked with a suture as a landmark for trimming. The 12 o'clock position was marked with a suture and after enucleation, the eyeball was immersed for 48 hours in Davidson fixative (35% distilled water, 20% formol (4%), 10% glacial acetic acid, and 35% absolute ethanol). Then eyeballs were kept 24 hours in 4% formaldehyde and ethanol 70%. Dehydration was carried out through successive baths of ethanol at increasing concentrations until clearing with xylene in an automatic tissue processor. Samples were embedded in paraffin taking into account the sample orientation and 4 *μ*m slides were obtained using a microtome. Optic nerve appeared in all cuts, so that they would be comparable between one another. Sections were then stained with Harris's hematoxylin stain (Polysciences Inc., Warrington, PA) and eosin following the standard procedure.

### 2.5. ONL and Neurosensory Retina Thickness and Photoreceptor Counting

ONL and neurosensory retina thickness measurements and photoreceptor counting were performed on hematoxylin-eosin stained sections. Images of slides were captured digitally with standardized microscope and camera settings. For ONL and neurosensory retina thickness quantification, a screen associated photomicrograph system (DSL-1 Sight, Nikon) was used. Photoreceptor counting was performed manually in 1,000x digital photographs. In order to standardize all tissue sample locations, four measurements (two in the upper and two in the inferior retina) were performed in each preparation, 1,000 *μ*m from the optic nerve for each study variable. Measurements were made by personnel unaware of the study groups.

### 2.6. Immunohistochemistry for Caspase-1 and Caspase-3

After pretreatment with antigen retrieval (DAKO) for 20 minutes at 95°C, paraffin-embedded sections were examined for immunohistochemical expression of caspase-3 and caspase-1. Caspase-3 antibody (Promega G7481) was used at a concentration of 1 : 100 and caspase-1 antibody (Millipore 92590) at a concentration of 1 : 250. Both antibodies were visualized with an anti-rabbit secondary antibody detection system (*Envision*, Dako). All reactions were revealed by diaminobenzidine (DAB) and counterstained with hematoxylin. As positive control for caspase-3 and caspase-1 detection, rabbit ovarian and lung samples were used, respectively. Negative control experiments included nonimmune serum of the same species as the primary antibody at the same protein concentration and incubation in buffer alone.

### 2.7. Determination of Oxidative Stress: TBARS and DHE

For lipid peroxidation (LPO) measurement, we slightly modified the method described by Conti et al. [25]. Thiobarbituric acid reactive substances (TBARS) were measured in retinal homogenates as indicators of lipid peroxidation [26, 27]. Diethylthiobarbituric acid-malondialdehyde (DETBA-MDA) complex was determined by fluorescence with 540/590 nm excitation/emission wavelength and all samples were measured in triplicate. The protein concentration was determined using a modified Bradford assay (Bio-Rad, Hercules, CA, USA) as we have used previously [26, 27]. As a second method to detect the presence of oxidative stress, in situ superoxide generation production was detected by fluorescence with dihydroethidium (DHE) (Molecular Probes). Dehydrated paraffin samples were incubated with DHE (125 mg) in a light-protected humidified chamber at 37°C for 30 minutes. The cell nuclei were labeled with TOPRO-3. The DHE images were obtained with a laser scanning confocal imaging system (Zeiss LSM-510 Meta) with a 585 nm long-pass filter.

### 2.8. Determination of the Total Antioxidant Capacity

The total antioxidant capacity (TAC) is a measurement in moles of antioxidant substances and determines the capacity of neutralization of free radicals. TAC is a sensitive and reproducible marker to detect changes in oxidative status, which often cannot be determined by measuring the antioxidants separately. For the present work we measured the TAC following the manufacturer's instructions (Total Antioxidant Power kit, Oxford Biomedical, Oxford, UK) to determine TAC based on the ability of antioxidants to reduce Cu^++^ into Cu^+^ in retinal homogenates.

### 2.9. Oxidative Ratio

In order to quantify the oxidative status of retinas, we calculated an oxidative ratio using data from oxidation (TBARS) and TAC using the following formula: TBARSx100/TAC.

### 2.10. Statistical Analysis

Values are reported throughout as the mean ± standard deviation (SD). Statistical significance was determined applying an analysis of variance (ANOVA) or a Kruskal-Wallis test to assess differences among groups. After a significant ANOVA, comparisons between groups were made with the following orthogonal contrasts: (1) control versus immediate sacrifice after exposure to light exposure; (2) control versus sacrifice one week after light exposure; and (3) immediate sacrifice after light exposure versus sacrifice one week after light exposure.

After a significant Kruskal-Wallis, a Mann-Whitney test was applied to analyze differences. Statistical significance was accepted at the 95% confidence level (*P* < 0.05), and analysis was performed by using the computer program SPSS (v. 15.0, SPSS Inc., Chicago, USA).

## 3. Results

### 3.1. Neurosensory Retina Thickness

**Figure 1:**
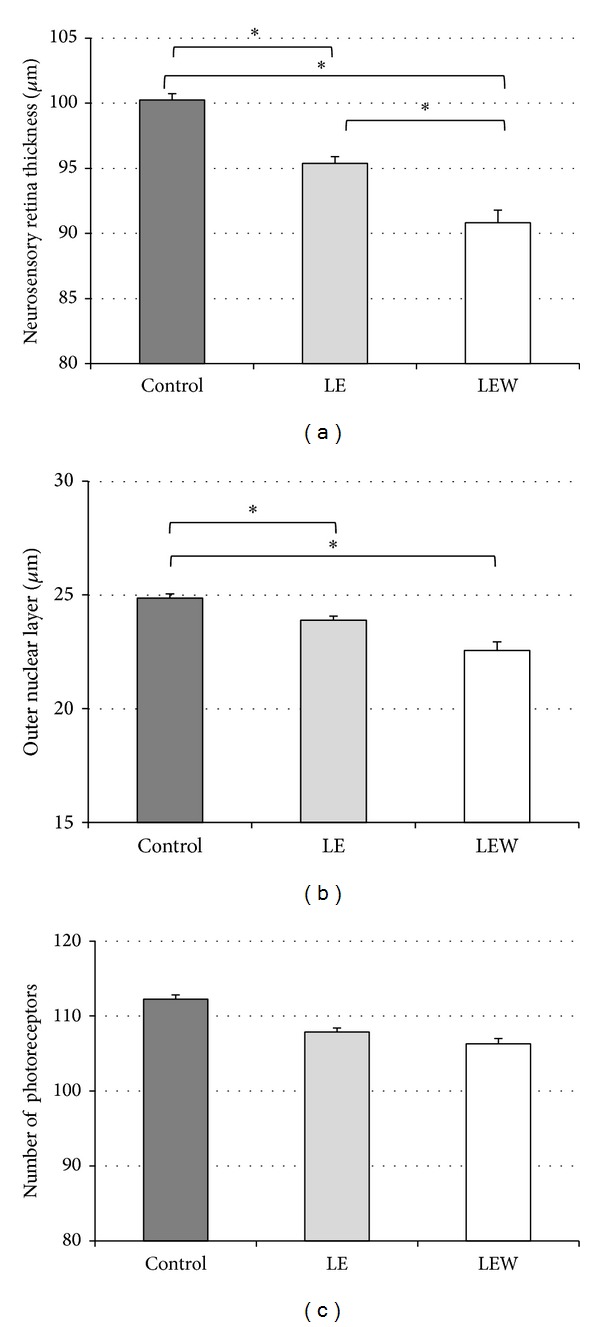
The effect of phototoxicity on The effect phototoxicity on retinal thickness and number of photorreceptors. (a) The thickness of the neurosensory retina in exposed animals is lower than the control group. Further, the LEW group neurosensory retina thickness is lower than in the LE group (*P < 0.05). (b) Thickness of the outer nuclear layer in the exposed rabbits was significantly lower than in control group. (c) There were no significant differences in the number of photoreceptors in any group. Results are expressed as mean ± SEM.

ONL and neurosensory retina thickness measurements and photoreceptor counting were performed on hematoxylin-eosin stained retinal sections. We observed a significant decrease in neurosensory retina after light exposure. The thickness of the neurosensory retina in the LE group (95.37 ± 4.56 *μ*m) and LEW group (90.81 ± 8.14 *μ*m) was lower than the control group (100.25 ± 4.31 *μ*m) (*P* = 0.041 and *P* = 0.003, resp.). Furthermore, in the LEW group the neurosensory retina thickness was lower than in the LE group (*P* = 0.049). Representative images from all study groups are shown in Figure 1(a).

All the histological findings are summarized in Table 1.

### 3.2. Thickness of Outer Nuclear Layer

We found a large data dispersion, particularly in the LEW group. However, thickness of the ONL in the LE group was significantly lower than in the control group (23.89 ± 1.31 *μ*m versus 24.85 ± 8.4 *μ*m, *P* = 0.047). Similarly, the LEW group showed significantly lower ONL thickness compared with the control group (22.56 ± 2.85 *μ*m versus 24.85 ± 8.4 *μ*m, *P* = 0.007). Although the differences between the LE and LEW groups did not reach statistical significance (*P* = 0.076), data suggest that the thickness of the ONL decreases as the time between exposure to light and sacrifice increases (Figure 1(b)).

### 3.3. Number of Photoreceptors

**Figure 2:**
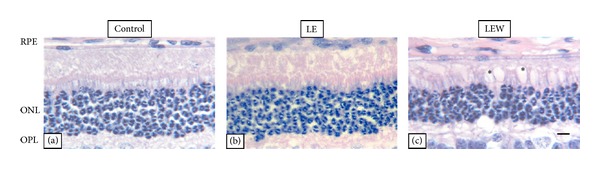
Hematoxylin-eosin stained retinal cross sections. (a) Control group, (b) LE group, and (c) LEWgroup. An increase in vacuolation inside the outer segments of the photoreceptors of rabbits sacrificed one week after light exposure was observed in this study ((c), asterisk, scale bar: 50 *μ*m). (ONL) Outer nuclear layer, (OPL) outer plexiform layer, (RPE) retinal pigmented epithelium.

Despite the decrease in ONL, we found no statistically significant differences in the number of photoreceptors between the groups (*P* = 0.513), Figure 1(c). However, animals from the LEW group showed an increase in vacuolization inside the outer segments of the photoreceptors which was absent in the other groups (Figures 2(a)–2(c)).

### 3.4. Immunohistochemistry

**Figure 3:**
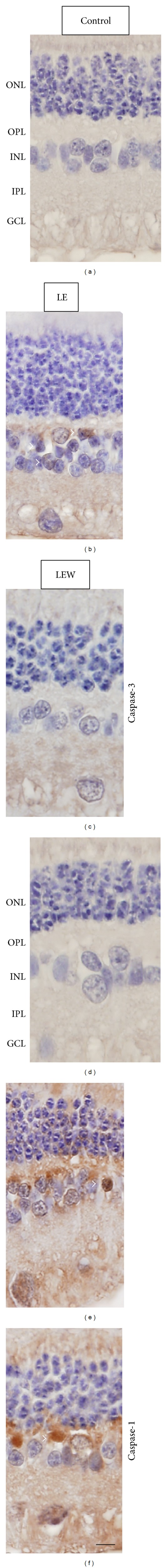
Phototoxicity induces apoptotic death and activation of inflammatory processes. ((a)–(c)) Caspase-3 immunohistochemistry showed no staining in control (a) and LEW rabbit retinas (c); however we found positive caspase-3 cells in the INL in LE rabbit retinas ((b) arrowheads). ((d)–(f)) Caspase-1 immunohistochemistry. Control rabbit retinas showed no caspase-1 staining (d). Labeling was seen (arrowheads) in LE (e) and LEW rat retinas (f). Scale bar: 50 *μ*m. Retinas were contrasted with hematoxylin. (ONL) Outer nuclear layer, (OPL) outer plexiform layer, (INL) inner nuclear layer, (IPL) inner plexiform layer, and (GCL) ganglion cell layer.

In order to detect the presence of apoptosis induced by light exposure, we assessed the presence of anti-caspase-3 by immunohistochemistry in retinal sections. The activity of caspase-3 was not detectable in the control group, Figure 3(a). We found caspase-3 activity in the inner nuclear layer in rabbit retinas from the LE group (Figure 3(b)). Caspase-3 activity then disappeared 1 week later in the LEW group (Figure 3(c)). On the other hand, caspase-1 activity, a protein involved in inflammatory processes, was not detectable in control animals (Figure 3(d)). However, cells in the inner nuclear layer were found to be caspase-1 positive in animals sacrificed immediately after exposure, LE (Figure 3(e)) and remained positive one week after light exposure, LEW (Figure 3(d)).

### 3.5. Biochemical Determinations

#### 3.5.1. Lipid Peroxidation Measured by TBARS

We found a significant increase in lipid peroxidation immediately after light exposure. The LE group had a statistically significant increase of TBARS when compared with the control group (5.24 ± 1.25 nmol/mg versus 4.65 ± 0.45 nmol/mg, *P* = 0.011). On the other hand, the TBARS value of the LEW group was significantly lower than the control group (3.91 ± 1.36 nmol/mg versus 4.65 ± 0.45 nmol/mg, *P* = 0.009) and they were also lower than the LE group (3.91 ± 1.36 nmol/mg versus 5.24 ± 1.25 nmol/mg, *P* < 0.001). These results are represented in Table 2. Values from all the biochemical findings are summarized in Table 2.

#### 3.5.2. Determination of the Total Antioxidant Capacity and Oxidative Ratio

**Figure 4:**
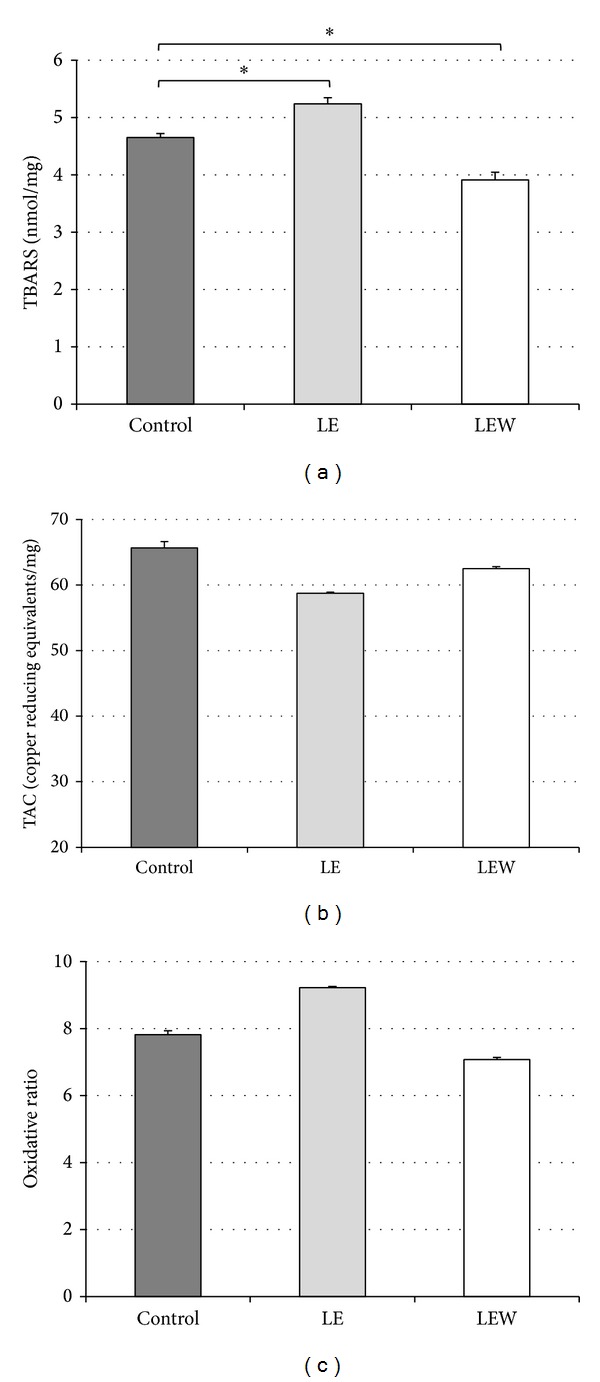
The effect of phototoxicity on TBARS, TAC, and oxidative ratio. (a) The LE group had a statistically significant increase of TBARS when compared with the control group (a). However, TBARS value of LEW group was lower than both the LE and the control group. *P < 0.05. There were no significant differences in the TAC or oxidative ratio in any group ((b), (c)). Results are expressed as mean ± SEM.

TAC, a measurement of antioxidant substances, was performed to determine the capacity of neutralization of free radicals. Further, in order to quantify the oxidative status of retinas, we calculated an oxidative ratio using data from oxidation (TBARS) and TAC. However, there were no statistically significant differences in the TAC (*P* = 0.635) or in the oxidative ratio between the studied groups (*P* = 0.635). These results are shown in Figures 4(a)–4(c) and Table 2.

#### 3.5.3. Detection of Superoxide Production by DHE

**Figure 5:**
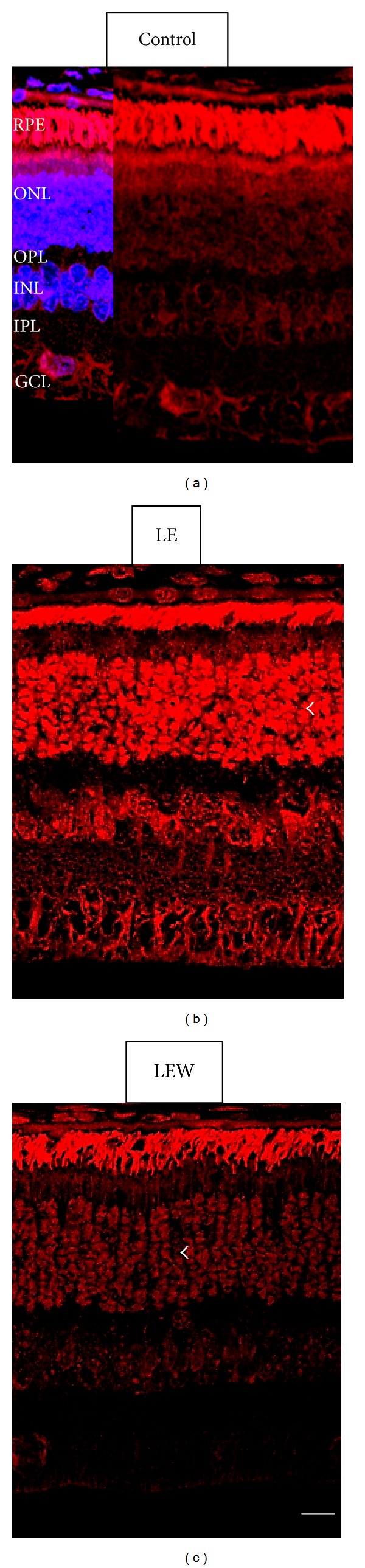
Superoxide generation was assessed in rabbit retinas with the fluorescent indicator DHE (red). (a) Confocal microscopic image of a rabbit retina from the control group. (b) DHE was detected in the LE group (arrowhead) at the ONL. (c) DHE fluorescence in the retinas from the LEW group was significantly lower. Staining of cell nuclei was observed for TO-PRO-3 (blue). Arrows indicate the ONL where the DHE was strong. Scale bar: 50 *μ*m. (ONL) Outer nuclear layer, (OPL) outer plexiform layer, (INL) inner nuclear layer, (IPL) inner plexiform layer, and (GCL) ganglion cell layer.

Likewise, retinal levels of superoxide were determined with DHE staining. DHE was absent in the control group (Figure 5(a)) but was strongly detected in the LE group, mainly in the outer nuclear layer, inner nuclear layer, and ganglion cell layer (Figure 5(b)). However, its presence was significantly lower in the LEW group in the aforementioned layers (Figure 5(c)), which confirms the initial increase of oxidative stress following light exposure that decreases when the animal is sacrificed one week after, observed with TBARS.

## 4. Discussion

In the present study, and for the first time to our knowledge, we describe some of the immediate and later biochemical changes associated with pathological exposure to light while developing a model of retinal phototoxicity in rabbits. Moreover, some histological changes observed were in accordance with previous data from other authors [28]. Retinas from rabbits exposed to light showed higher levels of lipid peroxidation and a decrease in ONL and neurosensory retina thickness.

Although some controversy exists over the role of phototoxicity in the pathogenesis of AMD, epidemiological evidence suggests a direct relationship between cumulative light exposure and the development and progression of this disease [9–13]. Consequently, there has been an increased interest in studying the pathologic effects of light on the retina and therapeutic strategies to prevent it, such as antioxidants and the use of blue light filtering IOLs [10, 13].

Retinal phototoxicity models in small rodents have been used in the majority of studies [12–14]. However, the use of larger animals, like rabbits, offers the advantage of having bigger eyes that enable the insertion of IOLs and studying the effect of this surgery in the retina as well as the possible effect of blocking blue and other visible light sources [22].

An increase in vacuolization inside the outer segments of the photoreceptors of rabbits sacrificed one week after light exposure was observed in this study. This finding was described by Grimm and Mukai and seems to be related with areas of minor and reversible damage, not sufficiently intense to activate the apoptotic cascade [29, 30].

Some studies demonstrate that retinal degeneration continues for several weeks after exposure to light [14, 16, 31] suggesting that an animal with a longer time period between the end of exposure and sacrifice will have more time to produce activation and operation of various mechanisms of damage as well as tissue regeneration. Our results agree with this hypothesis, given that histological damage continues after light exposure. We found a greater decrease in neurosensory retina and ONL thickness in the group of rabbits sacrificed one week after the light exposure compared with the group immediately sacrificed. Further, caspase-1 remained active in the retinas one week after light exposure. Distinct inflammasomes may upregulate caspase-1 which, in the macrophage cytoplasm, cleaves pro-IL-1*β* to active IL-1*β*, increasing inflammation and expression of proinflammatory genes [32]. Our study shows high caspase-1 levels 1 week after exposure, which is in line with the observation that macrophages are observed in the retina weeks after phototoxic stimuli (McKechnie and Foulds). Both findings suggest a long-term response by the mononuclear phagocyte system. In contrast, caspase-3, an important effector of apoptosis, was only detectable in the animals sacrificed immediately after exposure.

Increased levels of retinal lipid peroxidation upon exposure to light are well documented in different animal models including rabbits [12–14, 33]. Dzhafarov exposed rabbits with diabetic retinopathy to bright light and observed an acute increase in retinal lipid peroxidation [33]. In our study, the level of oxidative damage measured by TBARS in animals sacrificed immediately after light exposure was 12% greater than the unexposed group. However, one week after light exposure, lipid peroxidation was recovered to levels even lower than the control group, suggesting that some restoring mechanisms could have been activated in response to light damage. We could not find any study in the literature describing retinal levels of lipid peroxidation after a long period from the acute exposure to light. In addition, DHE results in this study confirm the initial increase in oxidative stress that decreases one week after light exposure. Our results suggest a recovery in oxidative status, which could respond to an increase in the antioxidant defense mechanisms that counteract retinal oxidative stress. However, we have not been able to confirm this theory because the levels of TAC in the three groups were not significantly different. TAC measurement includes the activity of various antioxidants present in a tissue, but not all. In general, it measures primarily low molecular weight antioxidants and chain breakers, excluding antioxidant enzymes. Other authors have found increased levels of superoxide dismutase or glutathione peroxidase in eyes of various animals exposed to light [34]. It would be very interesting to measure these enzymes in the retinas of rabbits exposed to light and analyze their variation after one week of recovery from light exposure.

Along with other authors, we believe that the initial step resulting in retinal damage is an acute increase in lipid peroxidation following light exposure, which damages photoreceptors and other retinal cells that ultimately induce their own apoptosis [13, 35]. Our study supports this by the presence of caspase-3. After light exposure, we found initially high levels of lipid peroxidation that decreased over the course of one week relative to controls. During this time, either the cells may be destroyed via apoptosis or they remain alive but show signs of damage such as vacuolated outer segments, if oxidative damage is not intense enough. We hypothesize that lipid peroxidation may decrease following an oxidative insult as the cell overcompensates its antioxidant efforts to counteract such an insult. Despite this rigorous antioxidant effort by the cell, the damage may be too great and continue its course, and progressive destruction leads to the greatest loss of ONL thickness observed at one week after exposure.

As the role of melanin is controversial [36–38] and appears to depend on the intensity of light received, we decided to use albino animals. However, with high light intensity as we used in this study, melanin is able to generate oxygen free radicals [39]. It is possible that if we had used pigmented rabbits, melanin would have acted as another chromophore capable of causing more oxidative damage. It is also possible that we might have found increased retinal destruction if we would have used elderly animals, as antioxidative mechanisms decrease with age [40] and lipofuscin concentration in the retina increases [41, 42]. However, older animals might present a number of other conditions that may affect or alter the phototoxic retinal damage mechanisms [43].

## 5. Conclusions

In conclusion, in this study we demonstrate that light damage produces an increase in retinal oxidative stress immediately after light exposure that can be recovered by compensatory mechanisms. In spite of that recovery at a molecular level, some structural damage appears at a period of time after light exposure that could end in apoptosis phenomena. Oxidative stress and inflammation are crucial in degenerative diseases of the retina; this is particularly interesting for AMD, a disease in which these factors have been implicated as major players. Furthermore, this model may be useful in the future to study the protective effect against phototoxic damage of antioxidant substances or new IOLs with a yellow filter.

## Figures and Tables

**Table 1:** Histological results.

	*N*	Thickness of the outer nuclear layer (*μ*m)	Neurosensory retina thickness (*μ*m)	Number of photoreceptors
Control	6	24.85 (±0.84)	100.25 (±4.31)	112.24 (±10.30)
LE	18	23.89 (±1.31)*	95.37 (±4.56)*	107.87 (±9.55)
LEW	18	22.56 (±2.85)^∗†^	90.81 (±8.14)*	106.30 (±12.08)

Data are expressed as mean ± S.D. Statistically significant differences from control are marked as *P < 0.05 and differences from the LE group are marked as ^†^ *P* < 0.05. LE: 120 minutes of light exposure and immediate sacrifice of the animal, LEW: 120 minutes of light exposure with sacrifice of the animal one week after exposure.

**Table 2:** Biochemical results.

	*N*	TBARS (nmol/mg)	TAC (copper reducing equivalents/mg)	Oxidative ratio
Control	13	4.65 (±0.45)	65.65 (±6.20)	7.82 (±0.73)
LE	46	5.24 (±1.25)*	58.76 (±1.87)	9.22 (±0.38)
LEW	33	3.91 (±1.36)^∗†^	62.50 (±2.91)	7.07 (±0.71)

Data are expressed as mean ± S.D. Statistically significant differences from control are marked as *P < 0.05 and differences from the LE group are marked as ^†^ *P* < 0.05. LE: 120 minutes of light exposure and immediate sacrifice of the animal, LEW: 120 minutes of light exposure with sacrifice of the animal one week after exposure.
